# Healthcare providers’ experiences of promoting health literacy in migrant women after pregnancy: a qualitative study

**DOI:** 10.1093/heapro/daaf162

**Published:** 2025-10-03

**Authors:** Marie Jubran Leksell, Ulrika Müssener, Kajsa André, Pontus Henriksson, Josefin Wångdahl

**Affiliations:** Department of Health, Medicine and Caring Sciences, Linköping University, 581 83 Linköping, Sweden; Department of Health, Medicine and Caring Sciences, Linköping University, 581 83 Linköping, Sweden; Department of Health, Medicine and Caring Sciences, Linköping University, 581 83 Linköping, Sweden; Department of Health, Medicine and Caring Sciences, Linköping University, 581 83 Linköping, Sweden; Department of Neurobiology, Care Sciences and Society, Aging Research Center, Karolinska Institutet, Tomtebodavägen 18 a, 171 77 Stockholm, Sweden; Department of Neurobiology, Care Sciences and Society, Division of Nursing, Karolinska Institutet, Alfred Nobels Allé 23, 141 52 Huddinge, Sweden; Department of Public Health and Care Sciences, Uppsala University, Husargatan 3, Box 564, 751 22 Uppsala, Sweden

**Keywords:** health literacy, organizational health literacy, health promotion, migrant, Sweden, healthcare

## Abstract

Low health literacy is linked to poorer health outcomes, higher mortality, and reduced quality of life. Forced migrant women may face disrupted health literacy upon arrival, leading to decreased healthcare seeking and delayed maternity care. As migrant reproductive health is a key public health priority, it is vital to understand how to effectively deliver postnatal health information. The aim of this study was to explore healthcare providers’ experiences of providing health information after pregnancy to women who have migrated to Sweden. A qualitative interview study was conducted, in which purposive sampling was used to recruit twenty healthcare professionals with a variety of occupational backgrounds and expertise. Inclusion criteria included having experience working with migrant women who had given birth. Data were analyzed using reflexive thematic analysis. Three main themes were identified in the study. These include building trust and confidence at the organizational level, providing health information in native languages through various settings, and adapting information to the varied needs and circumstances of the target group. Migrant women’s health literacy can be improved by fostering trust through continuous, culturally sensitive care that spans pregnancy and the postnatal period. Healthcare providers emphasized the need for personalized support and communication tailored to language, culture, and understanding. Organizational support such as interprofessional collaboration, continuity of care, and use of community-based resources was seen as essential. Providing information in native languages was identified as a key strategy for making health information accessible and actionable.

Contribution to Health Promotion

- According to the World Health Organization, health literacy is a critical component of health promotion.
- In this qualitative study, three main themes were identified as crucial elements in promoting health literacy for migrant women after pregnancy: building trust and confidence at the organizational level, providing health information in native languages through various settings, and adapting information to the varied needs and circumstances of the target group.
- Migrant women’s health literacy may be improved by fostering trust through continuous, culturally sensitive care that spans pregnancy and the postnatal period.

## BACKGROUND

As of 2024, there are ∼29 million non-EU citizens living in European Union countries (Eurostat 2024). Migrant health, including reproductive health, therefore represents an important public health priority. Several studies have shown significant health disparities between native-born and foreign-born women in terms of reproductive health. For instance, there is evidence that there are inequalities in the prevalence of adverse pregnancy outcomes such as gestational diabetes, birth complications and low birth weight children, although they may vary by maternal birth regions or ethnicity (Venkatesh *et al*. 2022, Shirvanifar *et al*. 2024, Xie *et al*. 2025). Importantly, the period after pregnancy has been identified as an important time to promote healthy lifestyle behaviors in the mother as weight retention and poor diet quality after pregnancy have been linked to cardiovascular disease risk factors later in life (Kirkegaard *et al*. 2021, Lai *et al*. 2023, Kramer *et al*. 2024). Thus, the promotion of healthy body weight and lifestyle behaviors after pregnancy may have long-lasting effects on maternal health.

Health literacy is an important determinant of people’s health (Magnani *et al*. 2018). Health literacy ‘*entails people’s knowledge, motivation and competences to access, understand, appraise, and apply health information in order to make judgments and take decisions in everyday life concerning healthcare, disease prevention and health promotion to maintain or improve quality of life during the life course’* (Sørensen *et al*. 2012, page 3). Low levels of health literacy have been consistently linked to poorer health outcomes, increased risk of mortality and decreased quality of life (Berkman *et al*. 2011, Magnani *et al*. 2018, Fan *et al*. 2021). Thus, both the World Health Organization and the American Heart Association have highlighted the relevance of health literacy as a priority for the prevention of non-communicable diseases (Magnani *et al*. 2018, World Health Organization 2022). Forced migrant women may experience a disruption to their health literacy upon their arrival in a new country (Mohr *et al*. 2023). Limited health knowledge during and after pregnancy is associated with negative health behaviors, reduced utilization of healthcare services, and negative outcomes for mothers and their children (Khan *et al*. 2024). This highlights an urgent need for healthcare systems to provide tailored care that aligns with individuals’ health literacy levels. It is therefore of great importance that organizations working with migrant women consider health literacy. An organization that equitably enables individuals to find, understand, and use information and services to form health-related decisions and actions for themselves and others is considered to have a high organizational health literacy (Santana *et al*. 2021).

The intersection of health literacy with digital literacy, cultural competence, and social determinants of health is not fully explored (Ahmadinia 2023), leaving gaps in understanding their combined impact on the health of migrant women. Furthermore, marginalized groups (e.g. migrant women) are often underrepresented in research (Pardhan *et al*. 2025), limiting insights into their specific needs and barriers. In Sweden, maternity and child healthcare is free of charge, with regular appointments both during and after pregnancy. Thus, healthcare providers such as midwives and child health nurses have an important role in providing information and supporting health and health literacy after pregnancy. However, previous research has shown that communication between healthcare providers and patients with migrant background is challenging, due to, for example, language barriers and cultural differences (Mårtensson *et al*. 2020, Bains *et al*. 2021). This suggests a need for a deeper understanding of healthcare providers’ experiences of delivering health information and thoughts regarding promotion of health literacy in the postnatal period to women with migrant background. The aim of this study was therefore to explore healthcare providers’ experiences of providing health information after pregnancy to women who have migrated to Sweden.

## METHODOLOGY

### Study design and setting

A qualitative interview study was conducted. Individual semi-structured interviews were considered appropriate for exploring health providers’ experiences and thoughts as they enable the participants to fully share their own experiences and knowledge regarding health information among migrant women after pregnancy (Braun and Clarke 2022, Byrne 2022). The study was conducted within the healthcare setting in Sweden including maternity clinics, healthcare centers, and public health units. Data were collected from four different healthcare regions in Sweden, each with a highly diverse population in terms of birth regions and educational backgrounds, encompassing both rural and urban areas.

### Participants

Purposive sampling was used to recruit healthcare professionals with a variety of occupational and professional experience and expertise. Inclusion criteria were healthcare professionals who had experience in health promotion for migrant women post childbirth. These inclusion criteria were set to recruit participants of various ages and experiences of working with health and migrants. Participants were recruited by MS (Maryam Shirvanifar, PhD student and dietician), AL (Alice Lindh, medical student) and KA (Kajsa André, medical student) and ML (Marie Leksell, PhD student, M.D). Others in the research team were two associate professors and one assistant professor with experience of qualitative methods and research in the field of health literacy, migration, and health promotion. Several team members also had experience from clinical and practical health promoting work with migrants (M.J.L., M.S., J.W., P.H.). Two of the researchers (M.J.L. and MS) are migrants with cross-cultural skills.

Forty-three professionals were invited to participate in the study. Five declined to participate due to lack of time, and 18 did not respond. The final study population therefore consisted of 20 healthcare professionals with a wide range of health professions (including midwife, dietician, medical doctor, and health communicator). The healthcare professionals’ ages ranged from 28 to 68 years, and they had between 2 and 38 years of professional experience (Supplementary File, Table S1). Eligible participants were informed about the study and confidentiality. Those who were interested in participating registered their interest by email and were then given information about the study by email.

### Dataset generation

Interviews were conducted to generate data for the study. All interviews were conducted in Swedish and performed individually by AL, MS, and K.A. during spring 2023. A total of nine interviews were conducted in person at a location selected by the participants and 11 interviews were conducted online through Zoom in a private setting. The interviews were recorded and lasted for about 1 hour (median: 57.5 min; range: 23–109 min). The participants did not have a relationship with the interviewers prior to the interviews. The interview guide was divided into three sections, health related work with migrant women postpartum, health literacy after pregnancy and questions related to an mHealth intervention (Supplementary File). This study focused only on aspects related to health literacy. In addition, basic background information such as the participants’ age, sex, profession, and years of experience in the profession was also recorded. The first interview served as a pilot test and worked as intended and was included in the study. As the initial interviews progressed, minor adjustments were made to deepen the exploration of health literacy through additional probing questions. Therefore, more probing questions about health literacy were asked after that. All interviews were included in the material and all recordings were transcribed word by word by a professional company. Emotions and pauses were not written down.

### Analysis

Data were analyzed using reflexive thematic analysis as described by Braun and Clarke (Braun and Clarke 2022), a flexible method that supports rich, detailed examination of the data and the development of themes and patterns through active researcher interpretation. The first author (M.J.L.) started by listening to the recordings and reading all the transcripts several times to understand the depth and breadth of the content, ensuring a complete view of the material. The data were analyzed on a semantic level to provide relevant results that could be useful for promoting organizational health literacy. M.J.L. thereafter systematically coded the dataset by identifying relevant sections or features related to the aim of the study. Once codes had been generated, potential themes were identified inductively by clustering similar codes together. Two co-authors (J.W., P.H.) took part in and commented on the suggested themes. Through discussions, alternative themes were iteratively developed and refined by M.J.L., J.W., and P.H. until the themes were clearly defined and the data meaningfully aligned with the theme to which it most strongly related. During the process, we continuously returned to the transcripts to ensure that the findings were grounded in the collected data.

### Ethics

Ethical approval was obtained from the Ethical Review Authority (reference number: 2022-06733-01). The respondents were informed orally and by email about the study aim. They were informed that participation was voluntary, and that they could interrupt the interview at any time. The respondents were also informed that their answers were confidential, and that no identifying information would be on the transcripts. In the case of quotations, no information about the respondent would be revealed. All participants gave consent before the interviews and agreed to have the interviews recorded.

## RESULTS

### Overview of the themes

**Figure 1.**
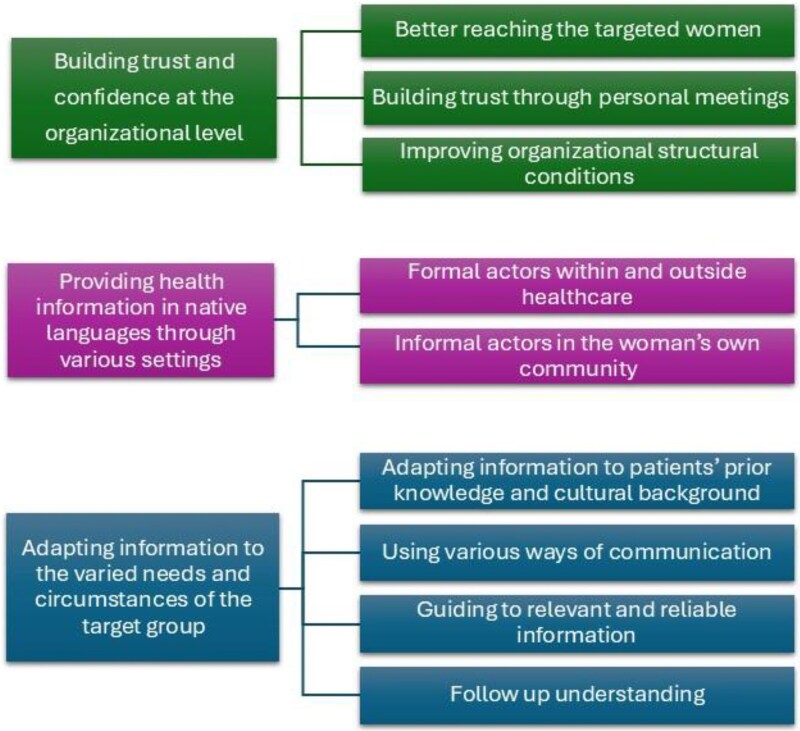
Overview of the themes and subthemes.

As shown in Fig. 1, three main themes were generated: (i) building trust and confidence at the organizational level, (ii) providing health information in native languages through various settings, (iii) adapting information to the varied needs and circumstances of the target group.

### Building trust and confidence at the organizational level

#### Better reaching the targeted women

Healthcare providers emphasized the importance of establishing meaningful connections with women, offering them the support and resources they need. Through active involvement, they could help women to access relevant information and services. Many participants stated that it was difficult to locate parents in need of help or information highlighting the need to create effective strategies for identifying such parents. The healthcare providers also stated that it would be of great value to keep focus on women both during pregnancy and the post pregnancy period. The migrant women then already had a relationship with the health providers and could easier be helped during this critical phase of motherhood. The healthcare providers also stated that it might be easier to reach women in the target group if they were able to have the same healthcare provider both during and after pregnancy.

I think that a midwife who has followed a woman through pregnancy also has an opportunity at the postnatal check-up to suggest different things, for example, to maintain weight or lose weight after childbirth (Healthcare provider J).

#### Building trust through personal meetings

The healthcare providers stated that healthcare meetings are a vital opportunity to build trust and create a meaningful connection with patients. Further, they stated that trust can be strengthened through physical visits, where healthcare providers take time to understand the individual’s background, including cultural differences. By actively asking about and considering cultural aspects when taking a medical history, providers can avoid stereotypes and make individual assessments that honor each patient's unique experience. Building trust was seen to strengthen patients’ confidence in the healthcare system.

And at the same time, it's a bit sensitive too, because you haven't met the woman before and then you have to start discussing these issues. So it's important to gain their trust before you can ask tough questions or decide what to say or what information to give (Healthcare provider A).

Healthcare providers also stated that in all kinds of healthcare, it is important to see and acknowledge the patient as an individual and not as a migrant group member, especially when working with women. Further they stated that it was of importance to motivate and highlight the woman’s abilities, reinforcing her strengths rather than focusing on limitations. The healthcare’s capacity to tailor individual coaching can encourage progress and help build the patient’s confidence in and trust in their healthcare journey. It also includes respecting the patient’s interpretation and ensuring they feel comfortable asking questions, fostering an environment where open communication can flourish.

We must respect their culture and them as individuals. As human beings. Each and every one of them. Just as we do with everyone else (Healthcare provider N).

The healthcare providers stated that scheduling follow ups and reminding patients about previous advice further strengthens the continuity of care and trust. By listening to the patient’s needs and being open to adjusting care based on those needs, healthcare providers can ensure that the care plan remains patient-centered. Focusing on the patient’s abilities, respecting their interpretation, and avoiding any form of diminishment were seen as key elements in maintaining a respectful and empowering healthcare experience.

That's something we could be much better at, continuity. That you see the same doctor, for example, in primary care, the same nurse and feel that there is time to listen and so on, that is very important (Healthcare provider I).

#### Improving organizational structural conditions

Some healthcare providers stated that improving healthcare conditions was a key factor in building trust between healthcare professionals and women. Several key areas were addressed. Strengthening staff in vulnerable areas was mentioned as essential to ensure that all patients receive the care they need. By avoiding staff shortages, continuity of care could be maintained, allowing healthcare professionals to build strong relationships with patients based on trust and consistent communication.

It is important to have time to follow up and ensure that the patient has understood and taken in the information we have provided. It is about providing support for change. It is much more complex than that, because there are so many different factors at play (Healthcare provider C).

Healthcare providers also stated that it is important to clearly explain their roles, ensuring that patients understand they are not part of social services. Interprofessional collaboration across different clinics and units was seen as key to delivering high-quality care. This includes the use of interprofessional and multidisciplinary teams, enabling collaboration across specialties, which enhances the overall healthcare experience for patients.

New parents must be able to trust that healthcare is a place that wants the best for them (Healthcare provider C).

Emphasizing the need for different teams to question their organization’s working methods was considered crucial for continuously improving care. Further, cultural competence was highlighted as a key factor in delivering equitable healthcare. By utilizing colleagues with language and cultural knowledge, healthcare providers can better engage with diverse patient populations and provide care that respects cultural differences.

For example, if I can speak in my language or my colleagues speak other languages, it is much easier to get the information across. We have several different languages at the clinic and that usually helps many women. They are also very relieved (Healthcare provider M).

### Providing health information in native languages through various settings

#### Formal actors within and outside healthcare

The healthcare providers stated that colleagues who speak the language could be used to facilitate communication, ensuring that patients feel understood and comfortable. Additionally, offering patients the ability to chat with staff who speak the same language can help with more immediate concerns or questions, making the healthcare experience smoother and more accessible. To further improve communication, it is essential to set higher standards for interpreters, so that proper information is given. Skilled interpreters play a crucial role in translating not just words but also the nuances of cultural context, ensuring that information is conveyed accurately and empathetically.

I have many colleagues who are very knowledgeable about languages, whereas I, for example, lack the ability to speak many languages. They can step in and help and assist me in explaining certain things (Healthcare provider B).

Healthcare providers highlighted the importance of employing health communicators who speak the target group's language to facilitate the dissemination of essential information. These can not only bridge the language gap but also build trust within the community. Further, they can be useful in educating patients on specific topics, offering targeted advice and information that resonates with the cultural and linguistic needs of the migrant community. In many communities, certain figures hold significant influence. The healthcare providers suggested different community interventions based on certain needs in the target group in various areas. One effective approach could be to meet outside of traditional healthcare settings, such as at open preschools or community centers. These familiar environments can make healthcare interactions less formal and more accessible, creating spaces where people feel comfortable asking questions and seeking support. The healthcare providers believed that including the community in healthcare initiatives would make an impact. Community involvement was seen to foster trust and ensure that the needs that are culturally sensitive and meaningful for the target group were addressed. Citizen offices and centers were also considered to play a key role in spreading important healthcare information. These local hubs, which are often deeply connected to the community, can help disseminate health messages, provide resources, and serve as points of contact for individuals seeking support.

Transcultural Center has health communicators. All of them have been newcomers themselves and are language carriers. And have a large network of newly arrived people. Who have cultural expertise in how to best convey information to a person who has a different cultural background than Sweden. It is an incredible resource (Healthcare provider B).

#### Informal actors in the woman’s own community

Healthcare providers stated that an effective way to share important information was by involving the migrant community. By getting help from this group healthcare providers could ensure that information reaches the intended audience more effectively and with greater trust. Arranging information sessions in the native language allows for clearer communication and better understanding, making it easier for individuals to absorb and act on the information provided. These sessions were seen by the participants to serve as a platform for questions, discussions, and knowledge sharing in a comfortable environment. Information can be further disseminated through the women’s closer networks, where individuals share in a more informal way useful information and resources. Informing each other was considered to build a strong, self-sustaining system of communication.

I think it could be linked to doulas and imams. They have been disseminators of information. They usually have high status within their own group. If the information comes from them, people will listen (Healthcare provider C).

To effectively reach and support diverse patient groups, healthcare providers believed that they could use key individuals as support, such as people from community associations. These key individuals often hold trusted positions and can facilitate communication between healthcare providers and the community. They play a crucial role in bridging communication gaps by sharing the same language and culture. Healthcare initiatives can be further enhanced by seeking help from associations and gatherings outside of healthcare. In addition, healthcare providers stated that utilizing actors outside of healthcare could help reach a larger target group. Communities often rely on social networks to share information quickly and effectively, allowing healthcare providers to tap into existing structures and arenas where information flows between individuals. Further, the participants reported that utilizing high-status individuals, such as imams or doulas, can greatly enhance outreach efforts, as these individuals are trusted and respected within their groups. They were seen to help convey messages more effectively and ensure that critical healthcare information is received and acted upon.

Once again, I'm a great believer in the idea that one's own countryman or countrywoman should go out with the information and explain. In this way it becomes a little more legitimate to listen. Because then you think that she is also from my country, and she knows this, and it is legitimate to absorb then (Healthcare provider O).

### Adapting information to the varied needs and circumstances of the target group

#### Adapting information to patients’ prior knowledge and cultural background

To provide personalized care, healthcare providers stated that they need to adapt information based on the patient's prior knowledge and cultural background. For instance, food-related information could be tailored to suit the patient’s cultural or regional preferences. Culturally adapted information was seen to ensure that the content not only reaches the audience but is also understood within the context of their cultural background. If the information aligns with the message in the native language, the healthcare providers believe that misunderstanding could be avoided. In situations where patients have limited literacy, healthcare providers stated that they need to consider the amount of information given, knowing that it must be memorized. This ensures that the information is tailored for easy absorption and does not overwhelm the patient. To provide information tailored to the target group, healthcare providers also thought that developing customized materials to meet individual pre-knowledge would be of great support. It was, for example, important to keep the language simple, avoid medical terminology and complex concepts and to offer easy-to-read materials. Further, written information could be complemented with practical advice, making the knowledge more applicable and actionable in real-world scenarios and possible to implement in everyday life.

How we can adapt it to easy-to-read Swedish, adapt certain questions, formulations, also not only foreign-born people but also for those with disabilities, that you can reach all groups (Healthcare provider F).

#### Using various ways of communication

Healthcare providers stated that patients absorb information in different ways. If necessary, interpreters should be used to overcome language barriers. To accommodate these diverse needs, they also suggested using a variety and combination of channels and tools to communicate important health information effectively. One effective method could be using images and basic visual information, which can make complex concepts easier to understand. For convenience, healthcare providers could use tablets to display visual content or refer patients to films as supportive material making the information more accessible, for example, YouTube videos and other visual resources that explain medical conditions or procedures. Healthcare providers believe that the use of various forms of information channels, such as reading, listening, and discussing makes the information more engaging and easier to retain.

While digital tools are common, some healthcare providers also emphasize non-digital channels for conveying information. For instance, they might use creative and hands-on methods like store tours or cook-along to teach patients about nutrition or lifestyle changes. These interactive experiences allow patients to learn through direct involvement, making the information more practical and applicable to daily life and in their health journey. To avoid overwhelming patients, the healthcare providers also believe that they need to avoid setting too large steps or goals when discussing treatment plans or health advice.

Cookalongs, where you show in different ways maybe, a lot on different levels, it feels like there is not a small thing, when you talk about healthcare, it has to be everything from the small, the shop walk, when you say it's small, to this a little broader, how to reach out to all your residents (Healthcare provider F).

#### Guiding to relevant and reliable information

The healthcare providers experienced that they were an important source for medical information for the women. Therefore, they believe that it was important that they give accurate information to ensure that patients are properly informed and able to make well-educated decisions about their care. The healthcare providers also stated that it is crucial that they help patients in choosing the most appropriate sources and methods for receiving information as well as referring them to credible sources. This could help the women to filter information and ensure that only relevant and important details are conveyed, and to prevent misinformation. Furthermore, they could also help patients find reliable information that aligns with their specific medical and personal needs. However, being able to do this required enough time for each patient, which was often lacking.

So here you really want to steer them to professional sites where you have scientific evidence for the information that you are providing. I think there are a lot of pitfalls that they can fall into (Healthcare provider A).

#### Follow-up understanding

Healthcare providers believed that it was important that patients not only receive information but also understand it and the reasoning behind the medical advice. One way was to review materials together with patients, explaining the content for better comprehension. Other ways included breaking down complex information into small, manageable amounts and offering interactive, dialogue-based sessions to promote questions and engagement. The Teach-Back method, where patients are asked to repeat back what they have learned was specifically mentioned and seen to ensure that key messages have been understood. The healthcare providers also believed that they need to summarize the provided information as it could help the woman to recall the most important content of the information. Asking follow-up questions and discussing related topics also helps gauge and reinforce understanding. Follow-up questions should be used during the ongoing visit as well as during the following visits. In cases where an interpreter was involved, special attention is given to ensuring that the patient comprehends the information, even when it was delivered in their native language.

Sometimes we think that we have given information and then you are satisfied, check on that. But that's not the same as having given information (Healthcare provider C).

## DISCUSSION

The study explored healthcare providers’ experiences of providing health information after pregnancy for women that have migrated to Sweden. Through the data three main themes were generated: (i) Building trust and confidence at the organizational level, (ii) Providing health information in native languages through various settings, and (iii) Adapting information to the varied needs and circumstances of the target group.

Our first theme ‘Building trust and confidence at the organizational level’ highlighted the importance of a multifaceted approach to healthcare delivery that emphasizes trust, cultural competence, and personalized care. Building trust includes strengthening staff in vulnerable regions to prevent shortages and maintain continuity of care, fostering strong patient-provider relationships, which agrees with previous studies (Sheppard *et al*. 2004, Haj-Younes *et al*. 2022). The need to build strong connections with women to ensure that migrant women receive the necessary support and resources has previously been highlighted as a key element for absorbing health information and taking health actions. This is supported by evidence indicating that trust between patients and healthcare providers may have an impact on patients’ adherence to treatment (Wu *et al*. 2022, Colombo *et al*. 2023).

The healthcare providers in our study stated that knowledge about the women’s cultural background was vital, with language- and culture-aware colleagues playing a key role in ensuring equitable and respectful healthcare for diverse populations. A previous study highlighted the importance of culturally and linguistically adapted information to ensure understanding and engagement since challenges include varying levels of prior knowledge and differences in learning preferences (Al-Adhami *et al*. 2021). Thus, cultural sensitivity and awareness of cultural diversity can establish trust between migrant women and the healthcare system which also was seen in our study. Based on this knowledge, we suggest that healthcare providers place greater focus on supporting migrant women after pregnancy by gaining cultural competence and making sure that a solid relationship is established. It may be of great value to take advantage of colleagues who share cultural background, both for exchanging knowledge and for making use of the existing resources. However, further research and initiatives in healthcare are needed to further explore how trust can be effectively established.

As described in our second theme ‘Providing health information in native languages through various settings’, the healthcare providers emphasized the importance of effective communication and outreach to better serve diverse patient populations. According to them, utilizing colleagues or health communicators who speak the same language as patients can significantly improve understanding, bridge knowledge gaps, build trust, and effectively share healthcare information. Indeed, Al-Adhami *et al*. (2023) showed in a study that civic communicators also providing health information in native language played a key role in bridging cultural and linguistic gaps, fostering trust, and making mental health discussions more accessible. Leveraging social networks within migrant communities can enhance health communication, particularly through native-language education and informal peer-led discussions among women (Mårtensson *et al*. 2020). The migrant’s own network can serve as an effective channel for disseminating health information, improving health literacy, and encouraging engagement with healthcare services. Further, research shows that informal support systems, such as women’s groups and community associations, play a pivotal role in helping migrant women navigate healthcare systems, especially in maternity care. The World Health Organization (2018) underscores the importance of culturally sensitive health systems and grassroots participation to foster trust and address barriers to care. This along with our findings suggest that native-language sessions and informal discussions within familiar social settings can significantly improve knowledge-sharing and promote equitable access to healthcare. Another implication from our study is that the healthcare system can gain from cooperating with formal and informal actors that speak the same language as the migrant woman to better reach the women.

In our final theme ‘Adapting information to the varied needs and circumstances of the target group’, healthcare providers emphasized the importance of tailoring information to patients’ prior knowledge and cultural backgrounds to provide personalized care. Language is also a crucial factor as identified in our second theme. Previous studies have shown that plain or native language may increase understanding and reduce misconceptions (Ayre *et al*. 2024, Ditshwane *et al*. 2024). This was also recognized by the healthcare providers in our study, especially when reflecting over women with limited literacy and their ability to absorb and apply medical information. Previous research also shows that language and cultural adapted interventions may improve health literacy, behaviors, and clinical outcomes (Fox *et al*. 2022). This may be reconciled with the results from our study, which suggest that customized materials and culturally sensitive communication may be prerequisites for effective communication and should be considered in clinical settings to promote health literacy after pregnancy among migrant women.

Our interpretation is that the three themes are linked to each other. Structural/organizational barriers in our first theme were considered by the healthcare providers in our study to affect the ability to have the time needed for each patient so the patient can receive the information in a personalized way according to his/her needs and conditions, and to follow-up given information. This is in line with previous research showing that healthcare providers are important channels for health information (Swoboda *et al*. 2018). In other words, healthcare providers are aware of what can facilitate for migrant women to access, understand, appraise, and apply information in a health promoting way, and they have a willingness to do so. However, if the structure of the organization does not allow this work it is problematic or impossible to tailor communication and information to the migrant women. This highlights the importance of organizational health literacy, which refers to the extent to which organizations equitably enable individuals to find, understand, and use information and services to make informed health-related decisions for themselves and others (U.S. Centers for Disease Control and Prevention 2024). This cannot be achieved if health literacy is not considered at all levels in the organization including the management level where structures and resources are chosen and decided.

Furthermore, the healthcare providers believed that language was an important factor for building trust, providing information and increasing understanding and making sure that the information is understood. This suggests that communication in the native language is of great significance (Tsai and Lee 2016, Pandey *et al*. 2022) and can be crucial when distributing health information for migrant women with limited Swedish linguistic competence. Although our findings are particularly relevant for promoting health literacy after pregnancy in migrant women, the fact that several of them have also been reported in previous studies on health information and migrants suggests that this knowledge may be transferable across different healthcare settings more broadly.

### Strengths and limitations

This study has both strengths and limitations. A strength is the qualitative design enabling healthcare providers to share their experiences and insights regarding the promotion of health literacy for migrant women after pregnancy. Further, through purposive sampling, the study ensured that participants had relevant experience in health promotion for migrant women post-childbirth. This gave rich data with different perspectives of health information related to migrant women and we believe that the information power is sufficient. The diversity of the study population, which included healthcare professionals from different regions in Sweden with various experiences, strengthens the transferability of the results to various healthcare settings. The use of the reflexive thematic analysis framework by Braun and Clarke (Braun and Clarke 2022, Byrne 2022) secured a structure and systematic analysis and the reporting guidelines for the same framework (Braun and Clarke 2024) helped us to be transparent and reflexive throughout the process. Regarding researchers’ reflexivity, both M.J.L. and J.W. have vast previous experience of working with migrants which has given them insight into both how healthcare works and how migrants experience the healthcare encounter. However, we were careful to continually go back to the raw data to ensure that we generated themes based on the participants’ statements.

A potential limitation was that the interview guide asked about experiences of promoting health literacy to migrant women. Health literacy is not a well-established concept in Sweden, and some healthcare providers may not have been familiar with the term. However, we do not think that this had had a great influence on our results since we explained the meaning of the term. Another limitation was that all healthcare providers in our study were women. However, this may not be a major limitation, as women make up a large proportion of Sweden’s healthcare workforce, particularly in patient-facing roles in reproductive and postnatal care. Furthermore, migrant women are a highly heterogeneous group, which the healthcare providers also highlighted in the interviews. The healthcare providers may have referred to very different groups of migrant women for example country of origin, language, culture, education level, and socioeconomic position, which is important to consider in future studies.

Finally, our findings reflect only the perspectives of healthcare providers, thereby motivating future research on migrant women’s own views on how to promote their health literacy after childbirth.

## CONCLUSION

This qualitative study of healthcare providers provides insights relevant for the promotion of health literacy after pregnancy in migrant women. First, the participants emphasized the importance of building trust and confidence for improved care. Second, they believed that providing health information in the patient’s native language, across various settings, may enhance the uptake of health information. Finally, the participants stressed that there are many ways to adapt health information to the needs of migrant women after pregnancy, including tailoring information, guiding to relevant information, and follow-up to ensure understanding. Although our study provided insights that are important for promoting health literacy in clinical care, structural barriers remain that need to be addressed.

## Supplementary Material

daaf162_Supplementary_Data

## Data Availability

In order to safeguard participant confidentiality, complete data (transcribed interviews and audio recordings) will not be made publicly available. Transcribed interviews may be available from the corresponding author upon reasonable request.
