# Supplementary material for: Healthcare providers’ experiences of promoting health literacy in migrant women after pregnancy: a qualitative study
Source: Health Promot Int. 2025 Oct 3;40(5):daaf162. doi: 10.1093/heapro/daaf162 (PMC12492001; doi:10.1093/heapro/daaf162)
Supplement: daaf162_Supplementary_Data [file daaf162_supplementary_data.pdf]

SUPPLEMENTARY MATERIAL

Healthcare providers' experiences of promoting health literacy in migrant women after pregnancy: a qualitative study

Marie Jubran Leksell[1], Ulrika Müssener[1], Kajsa André[1], Pontus Henriksson*[1,] Josefin Wångdahl*[2,3,4]

[1] Department of Health, Medicine and Caring Sciences, Linköping University, Linköping, Sweden

[2] Aging Research Center, Department of Neurobiology, Care Sciences and Society, Karolinska Institutet, Sweden

[3] Division of Nursing, Department of Neurobiology, Care Sciences and Society, Karolinska Institutet, Sweden

[4] Department of Public Health and Care Sciences, Uppsala University, Sweden

* These authors contributed equally to this work and are joint senior authors.

Corresponding author: Marie Jubran Leksell, Department of Health, Medicine and Caring Sciences, Linköping University, 581 83 Linköping, Sweden. E-mail: marie.leksell@liu.se Telephone number: +4613-28 10 00.

Interview Guide

I. Health promoting work in migrant women after childbirth

- How would you describe your role in working with migrant women regarding lifestyle and health?
- What responsibility do you think the healthcare system has in promoting a healthy lifestyle after childbirth?
- What opportunities does your professional role offer to promote healthy lifestyle habits after childbirth?
- What opportunities and challenges do you face in your professional role regarding the promotion of healthy lifestyle habits in migrant women after childbirth?
- In what ways is information and support offered to women to help them change their lifestyle after childbirth?
- How do you perceive the opportunities and challenges migrant women face in eating healthily and being physically active after childbirth?

II. Health literacy in migrant women after childbirth

- What role do you see the healthcare system playing in improving migrant women's ability to understand and use health-related information?
- What role do you think your professional role has in improving migrant women's ability to understand and use health-related information?
- Where do you perceive that women obtain health information?
- What opportunities and challenges do you perceive migrant women face in understanding health information?
- What challenges do you perceive that migrant women face in using the information you provide to them?
- What do you think could improve migrant women's ability to obtain and understand health information?

III. mHealth app in migrant women after childbirth

- What information and features do you think are needed in an app to promote a healthy lifestyle for migrant women after childbirth?
- What opportunities and challenges do you see with such an app?
- What is important to consider for the app to be functional linguistically and culturally for the women it is aimed at?

IV. Other

- Is there anything else you wonder about or would like to add?

Table S1. Descriptive characteristics of the healthcare providers included in the study

Health care provider	Health profession	Clinical experience (years)
A	Midwife	20
B	Healthcare administrator	4
C	Dietician	37
D	Midwife	26
E	Dietician	2
F	Healthcare strategy manager	8
G	Medical doctor	5
H	Psychologist	24
I	Dietician and operations manager	9
J	Specialist doctor	30
K	Midwife	15
L	Midwife	8
M	Midwife	6
N	Midwife	28
O	Manager for healthcare administration	38
P	Health communicator	15
Q	Psychotherapist	23
R	Health communicator	16
S	Psychologist	16
T	District nurse	12
